# On estimating the prevalence of use of medically assisted reproduction in developed countries: a critical review of recent literature

**DOI:** 10.1093/hropen/hoaa065

**Published:** 2021-02-17

**Authors:** Jasmin Passet-Wittig, Arthur L Greil

**Affiliations:** 1 Federal Institute for Population Research, Wiesbaden, Germany; 2 Division of Social Sciences, Alfred University, Alfred, NY, USA

**Keywords:** ART, developed countries, fertility treatment, help-seeking, infertility, impaired fecundity, medically assisted reproduction (MAR), prevalence, treatment-seeking, medical help to get pregnant

## Abstract

**BACKGROUND:**

Existing reviews on the prevalence of use of medically assisted reproduction (MAR) are relatively old and include mainly studies from the 1980s and 1990s. Since then, MAR has developed at a rapid pace, public awareness and acceptance of medical solutions to infertility problems has increased, and, consequently, the use of MAR has risen in developed countries.

**OBJECTIVE AND RATIONALE:**

This study provides a comprehensive overview of the state of research on the prevalence of MAR use in women and men, as well as a critique of methodology used in studies of the use of MAR, and suggestions for moving forward.

**SEARCH METHODS:**

Articles were located via the databases Academic Search Complete, Biomed Central, FirstSearch, Google Scholar, Medline, Health and Medical Collection, Medline and Social Science Citation Index using the key words ‘infertile’, ‘infertility’, ‘subfecund’, ‘subfecundity’, ‘treatment’, ‘help-seeking’, ‘service use’, ‘service utilization’, ‘ART use’ and ‘MAR use’ separately and in various combinations. The focus was on studies from developed countries, published between 1990 and 2018, in English, German or French.

**OUTCOMES:**

In this article, we have reviewed 39 studies covering 13 countries or regions; approximately half of these covered the USA. Ten studies were published in the 1990s, 10 in the 2000s and 19 since 2010. Studies report different types of prevalence rates such as lifetime and current prevalence rates of MAR use. Prevalence rates are based on very different denominators: women who tried to become pregnant for at least 12 months without success, women who experienced at least 12 months of unprotected intercourse without success, women of reproductive age from the general population or women with a life birth. There are few studies that report help-seeking rates for men or make direct comparisons between genders. Knowledge on medical help-seeking across different stages, such as seeing a doctor, undergoing tests, having operations to restore fertility or ART, has started to accumulate in recent years. There are conceptual reasons for being cautious about drawing conclusions about gender, regional, country level and differences over time in help-seeking rates.

**LIMITATIONS, REASONS FOR CAUTION:**

In a narrative review, the risk of bias in the interpretation of findings cannot be completely eliminated. The literature search was limited to languages the authors speak: English, French and German.

**WIDER IMPLICATIONS:**

In line with earlier reviews, we found that studies on help-seeking are not comparable across time and space, preventing researchers and healthcare providers from understanding the relation between social change, social policy, social structure and help-seeking for infertility. The discussion in this article should assist future researchers in designing better studies on the prevalence of MAR use. We provide suggestions for producing better estimates of the prevalence of MAR use. More cross-country and cross-gender comparisons are needed. Studies that treat help-seeking as a continuum and report on different stages are preferable compared to choosing arbitrary cutoff points, as is common practice in the studies reviewed.

**STUDY FUNDING/COMPETING INTEREST(S):**

None.

WHAT DOES THIS MEAN FOR PATIENTS?

Medically assisted reproduction (MAR), which refers to having medical help to become pregnant, has the potential to help people reach their parenting goals. Uptake of MAR has increased considerably since the birth of the first test-tube baby in 1978. Most people now accept the use of assisted reproduction and the treatments are broadly available. However, for various reasons, not everyone who is probably eligible for medical treatment actually uses it. It is important for healthcare providers to have estimates of the need for MAR and to understand if there are barriers to seeking MAR, so that they can find ways to reach all those who might benefit from their services.

Existing reviews of studies reveal that approximately half of infertile women use MAR and there are differences between countries in its use. However, these reviews are relatively old. Our review looks at information since 1990 from 39 studies covering 13 developed countries. Different ways of calculating rates of MAR use are examined critically and findings are discussed.

Overall, the results are not very reliable and are not comparable across time and countries for various reasons. For example, we found that studies use very different samples: some studies include only people with fertility problems, others include all women and men of reproductive age, and still others study only women who actually had a child. We found that there is a need for better studies, especially careful comparisons among countries, and between men and women. Also, researchers should report on use of the different stages of treatment (e.g. consulting a doctor, taking drugs, having IVF) rather than using an arbitrary cutoff point to determine if a person has used MAR.

## Introduction

Since the birth of the first ‘test-tube baby’ in the UK in 1978, ART)—as well as medically assisted reproduction (MAR) more generally—have developed at a rapid pace, creating increased faith in MAR, which includes all fertility treatments such as assisted inseminations, ovulation induction, surgery, IVF and ICSI (Zegers-Hochschild *et al.*, 2017). These developments have contributed to increasing uptake of MAR (Calhaz-Jorge *et al.*, 2017), which is possible because they are now broadly available in most developed countries.

The aim of this article is to provide a comprehensive overview of the state of research on the prevalence of use of MAR, a critique of conceptual issues in studies of the use of MAR, and identification of gaps in current research on this topic. Even though several studies have been published on this issue, existing reviews are relatively old (Schmidt and Munster, 1995; Boivin *et al.*, 2007). In 2007, Boivin *et al.* (2007) were able to report on 12 studies from more developed countries that met their inclusion criteria, but all of the studies reported were conducted in the 1980s and early 1990s. We now have 39 studies published since 1990 available for analysis. Furthermore, Boivin *et al.* (2007) summarized the findings of the studies they examined but did not offer a detailed examination of the huge conceptual problems in studies on MAR use or make recommendations about improving research on this subject. Better information regarding the prevalence of use of MAR is important for both theoretical and practical reasons. Theoretically, understanding use of reproductive medicine has implications for understanding the relation between social policy, social structure and medical help-seeking. Practically, it is important for healthcare providers to have estimates of the need for medical services in a population and to understand whether there are barriers to medical help-seeking so that they can find ways to reach all those who might benefit from their services. Furthermore, a critical discussion of conceptual problems of existing studies can aid future researchers in their attempt to conduct high-quality studies on the prevalence of use of reproductive medicine.

## Methods

Because the main purposes of this review are integrative rather than summative, we decided that a narrative review was more appropriate than a systematic review. Although systematic reviews are sometimes favored because they are more reproducible, they are usually narrower in scope than narrative reviews (Ferrari, 2015). Narrative reviews aim at synthesizing or integrating an area of research and moving it in new conceptual, theoretical, or methodological directions (Torraco, 2005, 2016; Elsbach and van Knippenberg, 2020). While there are very strict reporting standards for systematic reviews, standards for narrative reviews are less well established. In developing our methodological approach, we referred to the Scale for the Assessment of Narrative Review Articles for the quality assessment of narrative review articles (Baethge *et al.*, 2019) and to the guidelines spelled out by Torraco (2005, 2016).

Articles on the prevalence of MAR use were located via the data bases Academic Search Complete, Biomed Central, FirstSearch, Google Scholar, Medline, Health and Medical Collection and Social Science Citation Index using the key words ‘infertile’, ‘infertility’, ‘subfecund’, ‘subfecundity’, ‘treatment’, ‘help-seeking’, ‘service use’, ‘service utilization’, ‘ART use’ and ‘MAR use’ separately and in various combinations. In addition, we consulted the references cited and citing articles. Our goal was to locate any published source that provided at least one prevalence rate for MAR use based on the analysis of empirical data. Both authors participated in the literature search. In the case of disagreement between searchers, the source was included because we aimed at compiling as complete a list as possible. All sources were carefully scanned to determine whether they included an estimate of the prevalence of MAR use. If we found more than one source using the same data and providing the same prevalence rate, we counted only the first article published. We did not include sources that simply cited estimates based on other sources.

We limited our review to observational studies published in English, French and German. Our focus was on developed countries (United Nations, 2014) that are characterized by comparably low fertility and established economies. Over time, most of these countries can be said to have recognized that infertility is a relevant health issue, and some, but not all, currently provide some type of more or less general treatment coverage (IFFS, 2019). Differences among the countries included in this review still exist with regard to allowed methods, who are granted access to treatments and who may be reimbursed (Passet-Wittig and Bujard, 2021). However, comparability among developed countries is still greater than that which would be found in other world regions because of their more similar economic and demographic development.

This review covers all studies published in 1990 or later that we were able to locate and includes 39 studies using 30 data sets from 13 countries (USA, Canada, Australia, New Zealand, Denmark, Finland, France, Germany, Sweden, UK, Italy, Poland and Spain). We would like to emphasize that the period covered, namely 30 years, is extensive. Not only have reproductive technologies evolved considerably during this period, but the acceptance of MAR and its use increased. Moreover, countries have experienced considerable change in fertility behavior, most importantly the increasing age at first birth. This has to be taken into account when comparing prevalence rates of MAR use over time.

Table I provides an overview of the studies on the prevalence of MAR use. Table II provides an overview of the studies on the stages of medical help-seeking. In the tables, the findings from the papers reviewed are presented country by country. All tables include information on the sample, operational definitions of infertility and MAR use, and rates of MAR use. We paid careful attention to issues concerning the measurement and reporting of estimates of the prevalence of MAR use; other issues addressed by the sources were not evaluated. We had originally intended to include factors associated with MAR use (such as age, cultural norms, education, race/ethnicity, socio-economic status and social support), but space limitations prevented us from doing so. This topic will be discussed in a companion article.

**Table I:** Overview of studies on the prevalence of use of medically assisted reproduction (binary indicators) by region/country, sorted by year of publication (within countries).

Study	Data	Type of study, data collection, sampling, response rate (RR)	Year of data collection	Denominator	Numerator	Help-seeking rate	
						Current	Lifetime
**USA**							
**North America**							
Wilcox and Mosher (1993)	National Survey of Family Growth (NSFG)	Repeated cross section, personal interviews, random sample, RR: NA (*n* = 8450)	1988	Women 15–44 years with impaired fecundity (inability to have a child, physical difficulty conceiving/delivering a baby, pregnancy danger to women health, tried for ≥12 months) (*n* = 5.4 million, weighted)	Any infertility service	NA	43%
Greil and McQuillan (2004)	Own survey	Cross sectional, telephone interview, random sample, RR: 63% (*n* = 580)	2002	Women 25–50 years who ever tried ≥12 months to get pregnant or 1 year of unprotected intercourse (*n* = 196)	Consulted a doctor about infertility, any pregnancies were result of fertility treatment	NA	Overall: 39% With intent: 54% Without intent: 14%
Bitler and Schmidt (2006)	NSFG	Repeated cross section, personal interviews, random sample, RR: NA (*n* = 30 149, pooled)	1982, 1988, 1995, 2002 (pooled)	Women 15–44 years who ever had sex (*n* = 31 047)	Ever sought medical help to get pregnant, medical help to prevent miscarriage	NA	15%
Staniec and Webb (2007)	NSFG	Repeated cross section, personal interviews, random sample, RR: NA (*n* = 10 847)	1995	Women 22–44 years with unprotected intercourse for ≥12 months or who reported difficulties getting pregnant (*n* = 1210)	Ever sought medical help to get pregnant (prevention of miscarriage is excluded)	NA	31%
Anderson *et al.* (2009)	NSFG	Repeated cross section, personal interviews, random sample, RR: 78% (*n* = 4928)	2002	Men 15–44 years who ever had sex (*n* = 4109)	Ever sought help to become pregnant	NA	8%
Chandra and Stephen (2010)	NSFG	Repeated cross section, personal interviews, random sample, RR: NA (*n* = NA)	1995, 2002	Women 22–44 years with unprotected intercourse for ≥12 months or impaired fecundity (nonsurgically sterile, or physical problems to get pregnant or carry a pregnancy to term, or 36-month infertility (*n* = 1091 in 1995; *n* = 914 in 2002)	Ever sought medical help to get pregnant or to prevent miscarriage	NA	1995: 45% 2002: 39%
Duwe *et al.* (2010)	National Birth Defects Study (only control group with no birth defects)	Repeated cross sections, telephone interview, random sample from birth registers, RR: 69% (*n* = 5871)	1997–2004	Women in 10 surveillance regions who gave birth to a live infant with no birth defects (*n* = 5871)	Any medications or any procedures to help become pregnant for the last pregnancy	4%	NA
Hotaling *et al.* (2012)	NSFG (male sample)	Cross sectional, personal interviews, random sample, RR: 78% (*n* = 4928)	2002	Men aged 30–45 years (*n* = 2161)	Seen a doctor or other medical care provider to talk about ways to help have a baby	NA	8%
Simonsen *et al.* (2012)	Pregnancy Risk Assessment Monitoring System (PRAMS)	Repeated cross section, mixed mode survey, stratified sample of women who gave birth in eight states, RR: 75% (*n* = 25 358)	2004–2005	Women with live births who were trying to conceive (*n* = NA)	Assistance to become pregnant from a healthcare provider during the month of conception (all eight states)	NA	11%
Chandra *et al.* (2014)	NSFG	Repeated cross section, personal interviews, random sample, 2006–2010 RR: 77% (*n* = 22 682)	1982, 1988, 1995, 2002, 2006–2010	Women 25–44 years with current fertility problem (unprotected intercourse for ≥12 months or nonsurgically sterile, or physical problems to get pregnant or carry a pregnancy to term, or 36-month infertility) (1982: *n* = 32 055; 1995: *n* = 42 186; 2006–2010: *n* = 40 912) Men 25–44 years (2002: NA; 2006–2010: *n* = 40 917)	Ever used medical help to get pregnant or to prevent miscarriage	2002: 10% 2006–2010: 9%	1982: 42% 1995: 46% 2006–2010: 41%
Katon *et al.* (2014)	National Health Study for a New Generation of U.S. Veterans	Multi-wave panel, mixed mode survey, random sample, RR: 34% (*n* = 20 563)	2009–2011	Veterans, women (*n* = 620) and men (*n* = 2085) who ever experienced ≥12 months of trying to become pregnant without conception	Sought any help to get pregnant while trying to become pregnant	NA	Women: 43% Men: 39%
Sanders *et al.* (2014)	Pregnancy Risk Assessment Monitoring System (PRAMS)	Repeated cross section, mixed mode survey, stratified sample of women who gave birth in one state, RR: 81% (*n* = 9517)	2004–2008	Women with live births in Utah who were trying to conceive (*n* = 5238)	Received fertility treatments	NA	10%
Chin *et al.* (2015)	Furthering Understanding of Cancer Health and Survivorship in Adult (FUCHSIA) Women’s Study	Repeated cross section, telephone interview, representative survey, RR: NA (*n* = 1073; comparison women without cancer)	NA	Women who ever experienced 12 (ages 22–34) or 6 (ages 35–45) months of unprotected intercourse without conception (*n* = 278)	Ever visited a doctor or health professional for help becoming pregnant	NA	42%
Stanford *et al.* (2016)	Fertility Experience Study, Utah (includes a clinical sample and population sample; only latter reported here)	Cross sectional, mixed mode, random sample, RR: NA (*n* = NA)	2010–2012	Childless women 20–35 and married (2–5 years) with a history of primary infertility who tried ≥12 months to get pregnant at index date (*n* = 501)	Ever received medical treatment	NA	59%
Farland *et al.* (2016)	Nurses‘ Health Study II	Prospective cohort study, postal survey, sampling: NA; RR: 92% (*n* = 116 430)	1989–2009	Nurses 25–42 years at initial participation in 1989 who ever experienced 12-month period of trying to become pregnant without success (*n* = 7422)	Women who reported a cause or the cause was ‘not found’ on a question on the cause of their infertility	NA	65%
**Canada**							
Bushnik *et al.* (2012)	Canadian Community Health Study (CCHS)	Repeated cross section, RR: 89% (*n* = 29 858)	2009/2010	Couples were the women is 18–44 years who ever tried to become pregnant for >=12 months or had a baby (*n* = 4297)	Seen a doctor or medical care provider about problems conceiving	NA	15%
**Oceania**							
**Australia**							
Webb and Holman (1992)	Own survey	Random sample of women in Perth, personal interviews, random sample, RR: 90% (*n* = 1511)	1988	Married women 16–44 years who tried >12 moths to become pregnant (*n* = 47)	Sought medical treatment	49%	NA
Marino *et al.* (2011)	Own survey	Cross-sectional, personal interviews, cohort study of women born 1973–1975 in a hospital in Adelaide, RR: 49% (*n* = 974)	2005	Women 30–32 years who ever tried to conceive and reported difficulties getting pregnant never became pregnant, based on reproductive history (*n* = 159)	Ever sought assistance to help getting pregnant	NA	57%
**Europe**							
**Denmark**							
Schmidt *et al.* (1995)	Own survey	Cross-sectional, postal survey, random sample, RR: 78% (*n* = 2865)	1989	Women 25–44 years who ever attempted to conceive for >12 months without success (*n* = 418)	Ever sought medical examinations and/or treatment for infertility from a medical doctor or hospital	NA	47%
**Finland**							
Malin *et al.* (2001)	Own survey	Cross-sectional, postal survey, random sample, RR: 74% (*n* = 2189)	1994	Women 18–44 years who reported that they ever had difficulty becoming pregnant or having a child (*n* = 344)	Sought medical treatment	NA	67%
Terävä *et al.* (2008)	FINRISK survey	Cross-sectional, NA, random sample in six regions, RR: 76% (*n* = 4729)	2002	Women 25–64 years who had ever experienced a period of >12 months without conception or conception took >12 months (*n* = 701)	Ever had medical examination or use of fertility treatments	NA	57%
**France**							
Ducot *et al.* (1991)	Own Survey	Cross sectional, telephone survey, random sample, RR: 79% (*n* = 3181)	1988	Woman aged 18–49 who ever tried to conceive for 12 months without success (*n* = 387)	Either partner ever consulted a physician	NA	62%
Thonneau *et al.* (1991)	Own survey	Cross-sectional, questionnaire, exhaustive survey of practitioners in fertility clinics in three departments	1988/1989	Total population of women 19–44 years in France (*n* = 10 272 213)	Couples ever attending a public or private fertility clinic (projected based on numbers in two departments)	NA	14%
Moreau *et al.* (2010)	Own survey	Cross sectional, postal questionnaire, two-stage probability sampling, RR: 73% (*n* = 1616)	2000	Pregnancy attempts of women 18–60 who experienced ≥12 months of unprotected intercourse before a pregnancy leading to a live birth Women with no prior pregnancies (*n* = 508) Women who had a life birth (*n* = 827)	Consulted a physician because of difficulties in becoming pregnant	45% within 12 months, 75% within 24 months; 15% within 12 months, 22% within 24 months	NA
**Germany**							
Bruckert (1991)	Own survey	Cross sectional, personal interview, random sampling in West Germany and West Berlin, RR: NA (*n* = 2152)	1988	Married women (*n* = 99) and men (*n* = 110) with current unfulfilled desire to have a child where the woman was younger than 46	Seen a doctor or other consultant	Women: 60% Men: 31%	NA
Helfferich (2001)	Own survey ‘Frauen leben’	Cross sectional, telephone interview, random sample in three regions, RR: 44% (*n* = 1468)	1998	Women 20–44 years who ever experienced 12+ months of unprotected intercourse without conception (*n* = 219)	Specialist help for fertility problem	NA	44%
Helfferich *et al.* (2004)	Own survey ‘Männer leben’	Cross sectional, telephone interview, random sample in four regions, RR: NA (*n* = 1503)	2001–2004	Men 25–54 years with limited capacity to father a child or who ever experienced 12+ months of unprotected intercourse without conception (*n* = 261)	Seeking advice or medical help	NA	56%
**Sweden**							
Wulff *et al.* (1997)	Own survey	Cross sectional, postal survey, random sample of women living near a smelter or further away, RR: 67% (*n* = 960)	1992	Women 25–44 who ever experienced 12 months of unprotected intercourse without conception (*n* = 203)	Ever sought medical help to get pregnant	NA	49%
**UK**							
Datta *et al.* (2016)	National Survey of Sexual Attitudes and Lifestyles (Natsal-3)	Repeated cross section, personal interview, random sample, RR: 58% (*n* = 15 162)	2010–2012	Women (*n* = 923) and men (*n* = 470) 16–74 years who ever tried to conceive for > 12 months without success	Ever sought medical or professional help about infertility	NA	Women: 57% Men: 53%
**Multi-country studies**							
Olsen *et al.* (1996)	European Study on Infertility and Subfecundity (ESIS)	Cross-sectional, personal interviews, population-based samples in six countries/regions, Denmark (D): RR 87% (*n* = 1028), Germany (G): RR 54% (*n* = 1531), North Italy (NI): RR 74% (*n* = 1914), South Italy (SI): RR 79% (*n* = 815), Poland (P): RR 88% (*n* = 442), Spain (S): RR 70% (*n* = 900)	1991–1993	Women 25–44 years who ever experienced >12-month infertility (D.: *n* = NA, G.: *n* = NA NI: *n* = NA, SI: *n* = NA, P.: *n* = NA, S.: *n* = NA)	Ever sought any help because of problems getting pregnant	NA	All infertile (waiting time >12 months): D.: 51% G.: 43% Italy: 38% P.: 19% S.: 42% Planning pregnancy: D.: 62% G.: 57% I.: 51% P.: 37% S.: 53%

**Table II:** Studies on the stages of use of medically assisted reproduction by region/country, sorted by year of publication (within countries).

Study	Data	Type of study, data collection, sampling, response rate (RR)	Year of data collection	Denominator	Numerator	Help-seeking rate	
						Current	Lifetime
**North America**							
**USA**							
Wilcox and Mosher (1993)	NSFG	Repeated cross-section, personal interviews, random sample, RR: NA (*n* = 8450)	1988	Women 15–44 years with impaired fecundity (inability to have a child, physical difficulty conceiving/delivering a baby, pregnancy danger to women’s health, tried for ≥12 months) (*n* = 5.4 million, weighted)	Highest treatment stage: advice, infertility tests, specialized infertility service		10%, 9%, 24%
Staniec and Webb (2007)	NSFG	Repeated cross-section, personal interviews, random sample, RR: NA (*n* = 10 847)	1995	Women 22–44 years with unprotected intercourse for ≥12 months or who reported difficulties getting pregnant (*n* = 1210)^a^	Highest treatment stage: advice, infertility tests, ovulation medication, surgery, ART, no help	NA	6%, 7%, 11%, 3%, 6%, 67%
Chandra and Stephen (2010)	NSFG	Repeated cross-section, personal interviews, random sample, RR: NA (*n* = NA)	1995, 2002	Women 22–44 years with unprotected intercourse for ≥12 months or impaired fecundity (nonsurgically sterile, or physical problems to get pregnant or carry a pregnancy to term, or 36-month infertility) (*n* = 1091 in 1995; *n* = 914 in 2002)	Highest treatment stage (2002): sought advice or had testing only, ovulation or miscarriage, ART, insemination, or had a surgery to restore fertility, no help	NA	11%, 18%, 9%, 62%
Greil *et al.* (2010)	National Survey of Fertility Barriers (NSFB)	Two-wave panel, telephone interview, random sample, RR 54% (*n* = 4796)	2004–2007	Women 25–45 years who a) ever tried ≥12 months to get pregnant (*n* = 1045), b) ≥12 months of unprotected intercourse (*n* = 1117)	Type of help-seeking: considered treatment, talked to a doctor, tests, treatment, ART	a) Subfecund with intent (lifetime): 63%, 50%, 39%, 27%, 6%	b) Subfecund no intent (lifetime): 12%, 7%, 3%, 1%, .2%
Simonsen *et al.* (2012)	Pregnancy Risk Assessment Monitoring System (PRAMS)	Repeated cross section, mixed mode survey, stratified sample of women who gave birth in eight states, RR: 75% (*n* = 25 358)	2004–2005	Women with live births who were trying to conceive (*n* = NA)	Highest treatment stage (three states): ART, insemination and drugs, drugs only, other, none	NA	21%, 15%, 29%, 23%, 12%
Greil *et al.* (2013)	NSFB	Two-wave panel, telephone interview, random sample, RR: 54% (*n* = 4796)	2004–2007	Women 25–45 years who ever experienced 12 months of unprotected intercourse without conception in past 10 years (*n* = 1188)	Highest treatment stage: considered treatment, talked to a doctor, tests, other treatments, ART	NA	11%, 8%, 10%, 13%, 5%
Kessler *et al.* (2013)	NSFG	Repeated cross-section, personal interviews, random sample, RR: NA (*n* = 22 682)	2006–2010	Women 25–44 years, married or in a cohabiting relationship (*n* = 4558)	Highest treatment stage: infertility evaluation, evaluation and treatment		7%, 7%
Sanders *et al.* (2014)	Pregnancy Risk Assessment Monitoring System (PRAMS)	Repeated cross-section, mixed mode survey, stratified sample of women who gave birth in one state, RR: 81% (*n* = 9517)	2004–2008	Women with live births in Utah who were trying to conceive (*n* = 5238)	Highest treatment stage: ART, insemination, fertility-enhancing drugs, other medical treatment		18%, 14%, 41%, 11%
Stanford *et al.* (2016)	Fertility Experience Study, Utah (includes a clinical sample and population sample; only latter reported here)	Cross-sectional, mixed mode, random sample, RR: NA (*n* = NA)	2010–2012	Childless women 20–35 and married (2–5 years) with a history of primary infertility who tried ≥12 months to get pregnant at index date (*n* = 501)	Highest treatment stage: never received treatment, fertility drugs, assisted insemination, IVF	NA	41%, 26%, 19%, 14%
Crawford *et al.* (2017)	Behavioral Risk Factor Surveillance System (BSRSS)	Repeated cross-section, telephone interview, random sample, RR: 44% (*n* = NA)	2013	Women 18–50 years who ever tried to get pregnant for >12 months or with difficulties staying pregnant (*n* = 689)	Highest treatment stage: any treatment, consultation only	NA	50%, 18%
**Oceania**							
**Australia**							
Herbert *et al.* (2009)	Australian Longitudinal Study of Women’s Health, birth cohort 1973–1978	Multi-wave panel, postal survey, random sample, RR: NA (*n* = 14 247 in wave 1)	1996–2006 (4 waves pooled)	Women aged 28–33 who ever tried >12 months to conceive (*n* = 1031)	Highest treatment stage: advice, hormonal treatment or IVF	NA	35%, 36%
**New Zealand**							
Righarts *et al.* (2015)	Own survey	Cross-sectional, online survey, random sample of women in Otego and Southland, RR: 60.1% (*n* = 1125)	2011	Women aged 25–50 who tried to conceive without success for 12 months and/or sought help (*n* = 235)	Highest treatment stage: consulted doctor, consulted specialist, received treatment (only for first infertility episode)	61%, 41%, 33%	NA
**Europe**							
**UK**							
Templeton *et al.* (1991)	Own survey	Cross-sectional, postal survey, random sample in Aberdeen City District, RR: 86% (*n* = NA)	NA	Women 36–40 years (*n* = 157) and 46–50 years (*n* = 108) who ever experienced >2 years of trying or referral for investigation	Type of help-seeking: sought help from their general practitioner, were referred to hospital infertility services	NA	13% age 36–40, 7% age 46–50; 77% age 36–40, 62% age 46–50
Buckett and Bentick (1997)	Own survey	Cross-sectional, postal questionnaire, random sample in a region, RR: 85% (*n* = 548)	NA	Women 45–54 years who ever tried to conceive for >12 months (*n* = 126)	Highest treatment stage: general practitioner only, specialist appointment	NA	14 %, 34%
Oakley *et al.* (2007)	Own survey	Cross-sectional, postal questionnaire, two-stage-random sample, RR: 46% (*n* = 26 040)	2001	Women 40–55 years old at time of survey who ever tried to have a child or had a child (*n* = 6584)	Highest treatment stage: consulted a doctor about help getting pregnant, received fertility treatment	NA	16%, 8%

^a^Information on treatment stages is available only for *n* = 1091 women.

## Results

### Prevalence of MAR use

A still much-cited review of the prevalence of MAR use was written by Boivin *et al.* (2007). Based on 12 studies of medical help-seeking from more developed countries, this review reported the proportion of infertile women seeking any kind of medical care as ranging from 42 to 76%. On average, 42% sought medical advice and about 22% underwent medical treatment.

Prevalence rates for MAR use in the USA have been based mainly on the repeated cross-sectional National Survey of Family Growth (NSFG), which allows one to investigate time trends (Table I). According to the 1988 NSFG, 43% of women aged 15–44 years identified as having ‘current fertility problems’ ever sought MAR (Wilcox and Mosher, 1993). Two studies using NSFG data from 1995 reported lifetime help-seeking rates of 45% (Chandra and Stephen, 2010) and 31% (Staniec and Webb, 2007) among women identified as having current fertility problems. Both papers use a similar age group (22–44 years), but Chandra and Stephen use a definition of MAR use that includes prevention of miscarriage, whereas Wilcox and Mosher exclude treatment for miscarriage.

Furthermore, Chandra and Stephen (2010) reported a considerably lower percentage (39%) of help-seekers using the 2002 NSFG data and applying the same definitional criteria. They found that the difference in the rates for 1995 and 2002 is marginally statistically significant. A more recent publication based on the NSFG investigated trends over time by comparing the years 1982, 1995 and 2006–2010 (Chandra *et al.*, 2014). A peak of 46% was found in 1995 with somewhat lower rates in the other years. These differences, however, were not statistically significant. Interestingly, when comparing women without children over time, prevalence rates of MAR use decreased over time. The authors argue that this trend could be a reflection of postponement of first births to higher ages as women who try to have a child after 44 years are not part of the analysis.

The Utah Fertility Experiences Study (Stanford *et al.*, 2016) reported a much higher rate (59%) of MAR use for US women in 2010–2012. It includes only married childless women aged 20–35 years who were trying to have a child for at least 12 months at the time of the interview. The study inquired if they ever used MAR. A study by Farland *et al.* (2016) reports an even higher rate of MAR use of 65% among nurses 25–45 years old who tried to become pregnant for 12 months. In the absence of a direct question, MAR use was inferred based on a question on the cause of their infertility: if the cause was known it was assumed that medical help was sought.

A comparison of prevalence rates among women with intent (trying to become pregnant, 54%) and women without intent (okay either way with having a(nother) child, 14%) reveals how different the disposition for seeking MAR is among these groups (Greil and McQuillan, 2004). Yet another recent study reports a relatively high rate of ever seeking medical help of 42% among women with unprotected intercourse who may or may not have been intending to have a child (Chin *et al.*, 2015).

Instead of investigating the use of MAR among the infertile, some US studies have employed the full sample of women or men of reproductive age who are at risk for pregnancy. One study calculated the rate of MAR use among women aged 15–44 years who ever had sex at 15% (Bitler and Schmidt, 2006). Another study comparing women in the general population of the same age range found a slight decrease in use between 1995 and 2006–2010 (Chandra *et al.*, 2014).

Some US studies have taken a different approach by reporting rates of MAR use in samples of women with a live birth. Two studies used data from the Pregnancy Risk Assessment Monitoring System (PRAMS). They reported that 10–11% of women who were trying to become pregnant had sought treatment for infertility (Simonsen *et al.*, 2012; Sanders *et al.*, 2014). Yet another study of women with a live birth discovered that only 4% reported use of any fertility treatment (Duwe *et al.*, 2010).

We now turn to the few US articles which present rates of MAR use for men. In 2002, the NSFG included a representative sample of men aged 15–44 years for the first time. This can be considered an important innovation, as the overwhelming majority of articles on MAR use have surveyed women only. The NSFG medical help-seeking rate for all men who ever had sex was 8% (Anderson *et al.*, 2009); the rate was similar for slightly older men (30–45 years) (Hotaling *et al.*, 2012). Chandra and Stephen (2014) reported no change over time for men between 2002 and 2006–2010. Their analyses also revealed that the share of help-seekers is only slightly lower for men than for women. Katon *et al.* (2014) looked at rates of MAR use among a sample of men and women veterans serving during Operation Enduring Freedom/Operation Iraqi Freedom and also concluded that MAR use is lower for men. They found that the difference holds even in multivariable analyses using several socio-demographic controls. One reason for the gender differences discussed by the authors is that women may be more willing to disclose infertility-related information.

Information on MAR use is available for two other non-European English-speaking countries. Among Canadian women in a relationship who have tried to become pregnant or had a child, 15% have seen a doctor about problems procreating (Bushnik *et al.*, 2012). This rate is based on data from the Canadian Community Health Study (CCHS) from 2009/2010 among women aged 18–44 years. A study of a sample of young Australian women (aged 30–32 years in 2005) who have experienced difficulties reproducing reported a rather high rate of ever seeking medical help in becoming pregnant of 57% (Marino *et al.*, 2011). A current medical help-seeking rate of 49% among married women aged 16–44 years in Australia was reported by Webb and Holman (1992).

We now turn to findings from European countries. Most of these studies were conducted before 2000, and the rates of MAR use are therefore somewhat outdated. Based on the European Studies of Infertility and Subfecundity (ESIS), Olsen *et al.* (1996) reported rates of MAR use for five countries using two different bases for the calculations. Medical help-seeking rates using infertile couples who had been planning a pregnancy at the time of the infertility episode as the denominator were much higher than rates calculated using all infertile couples as the denominator (Denmark: 62% compared to 51%; Germany: 57% compared to 43%; Italy: 51% compared to 38%; Poland: 37% compared to 19%; Spain: 53% compared to 42%, respectively). A UK survey, the National Survey of Sexual Attitudes and Lifestyles (Natsal-3), asked women and men aged 16–74 years in 2010–2012 if they ever ‘sought medical or professional help about infertility’. As is the case in the USA, the rate of MAR use was found to be somewhat higher for women (57%) than for men (53%) (Datta *et al.*, 2016).

Rates of MAR use are relatively high in Scandinavian countries, possibly because of healthcare coverage in these countries. For Denmark, the lifetime prevalence of seeking fertility treatment among infertile women aged 25–44 years who had attempted to have a child was 47% (Schmidt *et al.*, 1995). In a study of a region in northern Sweden, Wulff *et al.* (1997) reported that 49% of the Swedish women with an extended period of trying to become pregnant sought medical help to reproduce. Among Finnish ever-infertile women aged 18–44 years in 1994, more than two-thirds (67%) of women who reported that they had had difficulty becoming pregnant or having a child had ever sought treatment (Malin *et al.*, 2001). Another Finnish survey using data from the FINRISK surveys of 2002 based their analysis on a slightly different sample (women aged 25–64 years who had experienced a period of more than 12 months without becoming pregnant) and found that 57% had ever had a medical examination or received fertility treatment (Terävä *et al.*, 2008).

Thonneau *et al.* (1991) used data from hospitals in three regions of France to obtain the number of women (19–44 years) treated for infertility after 1 year of trying and calculated a rate of MAR use based on the total population of the three regions. In the next step, this was extrapolated to the country level, resulting in an estimated percentage of receiving an infertility evaluation of 14% for the French population. Another French study estimated the rate of MAR use among women aged 18–49 years who ever tried to become pregnant for 12 months without success at 62% (Ducot *et al.*, 1991). Another French study estimated a rate of MAR use of 45% within 12 months for pregnancy attempts of women with no prior pregnancies and of only 15% for women who had a live birth before attempting another pregnancy (Moreau *et al.*, 2010). These rates, however, were based on a population of infertile women aged 18–60 years who had a live birth.

In Germany, Bruckert (1991) used data from women and men with a current unfulfilled desire to have a child who were married and where the woman was younger than 46 years; they reported a high current medical help-seeking rate of 60% for women and 31% for men. Helfferich (2001) reported a considerably lower lifetime medical help-seeking rate (44%) among ever infertile women 20–44 years of age in Germany. Both partners sought medical help in 37% of these cases, 55% of women sought medical help alone and in 8% of the cases only the male partner sought medical help. Unexpectedly, among German men with fertility problems, the lifetime medical help-seeking rate is 56% (Helfferich *et al.*, 2004), 12 percentage points higher than for women.

### Stages of MAR use

Information about progression through the stages of treatment derives mainly from US surveys. We find that studies that take such a more nuanced view are only now starting to accumulate: three papers were published in the 1990s, three in 2000, and nine since 2010. Studies differ in how many stages of MAR use are differentiated (e.g. talking to a doctor, taking tests, getting treatment) and in what types of treatments are considered (ovulation-inducing drugs, surgery, insemination, ART) (Table II).

Based on the 1988 NSFG, Wilcox and Mosher (1993) reported that in 1988 10% of infertile women 15–44 years mentioned seeking advice as their highest level of treatment, 9% had received tests and 24% had used specialized services. Staniec and Webb (2007) provided a more nuanced view using the 1995 NSFG data and reported that, among women with current fertility problems, 6% sought advice only, 7% had testing, 11% used ovulation-inducing drugs, 3% had surgery and another 6% had ART treatment. Using the 2002 NSFG, Chandra and Stephen (2010) found that, among women with current fertility problems, 11% ever sought advice or had testing only, 18% reported ovulation or miscarriage services as the highest level of treatment and 9% used ART, insemination, or had a surgery to restore fertility (see the previous section for a brief discussion of time trends in rates of MAR use based on the NSFG data). Based on the 2006–2010 NSFG, Kessler *et al.* (2013) reported that 7% of women aged 25–44 years with a cohabiting partner sought fertility evaluation only, and another 7% received treatment.

Greil *et al.* (2013) included an additional stage in their model and counted those who considered treatment but did not use it. Based on the National Survey of Fertility Barriers (NSFB) from 2004 to 2007, they found that 11% of ever infertile women considered treatment, 8% talked to a doctor, 10% had tests, 13% had treatments and 5% used ART. In another study, Greil *et al.* (2010) considered stages of treatment broken down by intention status. Among the ever subfecund with intent, 63% considered treatment only, 50% talked to a doctor but did not go further, 39% had tests but not treatment, 27% had conventional treatment only and 6% had ART. Medical help-seeking is considerably lower across all stages among the ever subfecund without intent; 12% considered treatment, 7% talked to a doctor, 3% had tests, 1% had treatment and 0.2% had ART.

Another US study using a random sample of childless, married women aged 20–35 years from Utah who had tried to reproduce for at least 1 year at index date reported that 26% used ovulation drugs, 19% mentioned use of assisted insemination and 14% used IVF (Stanford *et al.*, 2016). Crawford *et al.* (2017) used data from the Behavioral Risk Factor Surveillance System (BSRSS) from seven states that included the relevant questions on reproductive health in 2013. For women aged 18–50 years who ever tried unsuccessfully to reproduce, it was reported that 32% had no treatment, 18% had consulted only and 50% had received both consultation and treatment. Treatment in this case included drugs, IUI, ART, surgery, or other. High rates of treatment uptake are found across all treatment stages in studies of women who were actively trying to become pregnant and ultimately had a child (Simonsen *et al.*, 2012; Sanders *et al.*, 2014).

In Australia, among women aged 28–33 years who had experienced infertility, 35% had sought advice and another 36% had also sought treatment (Herbert *et al.*, 2009). For New Zealand, it was reported that, among women 25–50 years with current difficulties conceiving 61% consulted a doctor, 41% consulted a specialist and 33% received any treatment (Righarts *et al.*, 2015).

Information is available on the stages of medical help-seeking for only one European country: UK. A study from a rural area in the UK in the 1990s used data from women past their reproductive period (age 45–54 years) to calculate a real lifetime medical help-seeking rate (Buckett and Bentick, 1997). Among those who ever had experienced infertility, 14% had seen a general practitioner and 34% had visited an infertility specialist. Another UK study found that, among women aged 36–40 years who had tried unsuccessfully to become pregnant for at least 2 years, the overwhelming majority sought medical help: 13% consulted a general practitioner and 77% went to a hospital; among women aged 46–50 years, the shares were lower (7 and 62%) (Templeton *et al.*, 1991). Yet another UK study reports rates of medical help-seeking for all women at the end of their reproductive period (40–55 years): 16% reported ever consulting a doctor about problems conceiving, and 8% had received fertility treatment (Oakley *et al.*, 2007).

## Discussion

This review critically examined studies on the prevalence of MAR use. We showed that within the last 30 years considerable effort had been dedicated to answering the question of how many seek medical help. In this article, we have reviewed 39 studies covering 13 countries or regions. The central conclusion of this review is that it is not possible to answer this question in a satisfactory way: current studies are inconclusive, as they do not provide reliable information about MAR use in different populations. Consequently, we refrain from presenting averages or ranges of prevalence rates. We use the rest of this discussion to discuss central conceptual issues of studies on MAR use and provide avenues for further research. Table III gives an overview of aspects that we believe are crucial to consider in designing future studies.

**Table III:** Choices in designing studies of the prevalence of use of medically assisted reproduction.

Broad category	Specific category	Choice
Study design	Location of study	One country versus multi-country
	Respondent group	Women versus men versus both
		Individual versus couple
	Time focus	Current prevalence versus lifetime prevalence
	Type of study	Cross-section versus repeated cross-section versus longitudinal
Denominator for equation (population at risk)	Population	Infertile versus people of reproductive age
		Heterosexual individuals/couples, singles, same-sex individuals/couples
	Measure of infertility	Medical criteria versus self-perception or self-identification
		Infertile with intent versus all infertile
	Further sample specifications	Based on sociodemographic variables such as marital status, parenthood, education/occupation, age
Numerator for equation	Measure of treatment	Single cutoff point versus multi-stage measure
	Threshold for help-seeking	Talk to doctor versus tests versus treatment
		ART versus any treatment

### Conceptual issues

A well-defined numerator and denominator are required in order to calculate a prevalence rate of MAR use. The numerator displays the number of people seeking MAR; it is divided by the denominator, which reflects the size of the total population at risk of using MAR. Many of the problems with existing studies and the comparability of prevalence rates across time and space arise from definitional and measurement issues regarding the two core concepts: the population at risk and MAR use. Therefore, we begin with a discussion of these.

### Population at risk

From our review of the studies, we have identified three common denominators used to calculate prevalence rates of MAR use: women who tried to become pregnant for at least 12 months without success; women who experienced at least 12 months of unprotected intercourse without success; or women of reproductive age from the general population. All three of these numbers may provide useful information for professionals and policy makers, and it makes sense to measure all three. Depending on which measure is used for the denominators, the resulting help-seeking rates might vary considerably. As a general rule of thumb, rates based on the general population should be smallest, and rates based on samples of persons who tried to become pregnant should be largest.

We found few studies using women who have had a live birth as the denominator (Duwe *et al.*, 2010; Moreau *et al.*, 2010; Simonsen *et al.*, 2012; Sanders *et al.*, 2014). This is an approach we do not recommend because the women with a live birth are a highly selective group and thus of limited value for investigating medical help-seeking rates as not all women who sought medical help are included; that is, women who did not become pregnant or did not carry a pregnancy to term are excluded.

Some further comments are warranted with regard to the three possible denominators. If one is inclined to use the infertile as the basis for MAR use, the basic premise is that those who are infertile are the ones most likely in need of services. In this case, the core question is who should be considered as infertile. Most papers (Tables I and II) refer to the medical definition of infertility according to which a couple is considered to be infertile if they have failed to become pregnant after 12 months of regular unprotected intercourse (Zegers-Hochschild *et al.*, 2017), reasoning that the majority of women will become pregnant within a year (Gnoth, 2005). This definition is not clear regarding whether people are actually attempting a pregnancy. Not using contraception need not imply intent. In Europe, 43% of all pregnancies are unintended (Sedgh *et al.*, 2014). Against this background, it is understandable that some studies use women who were attempting a pregnancy without success and other studies use women who experienced a period of unprotected intercourse without becoming pregnant.

Very few papers that study infertile populations leave it up to the respondents to self-identify as infertile (Bruckert, 1991; Marino *et al.*, 2011). When self-reports are used to measure infertility, individuals are considered to be infertile if they state that it would be difficult or impossible for them to have a biological child. This is an interesting approach because women and men who perceive problems procreating are at risk for seeking medical help, regardless of the existence of a real medical problem (Passet-Wittig *et al.*, 2020). Interestingly, two US surveys, the NSFG and the NSFB, which were the basis for several papers reported here, combine medical considerations (i.e. 12 months infertility, miscarriages) with self-reports (Greil *et al.*, 2013; Chandra *et al.*, 2014). Going forward, then, population surveys should include questions about 12-month infertility, 12-month infertility among those trying to become pregnant and perceived fertility problems.

The general question that needs to be answered for each research project is who is considered to be at risk of using MAR services. Initially, in the development of reproductive medicine, the focus can be said to have been on helping infertile heterosexual couples become pregnant. This is perhaps reflected in the conceptual approach of many studies that focus on women who do not become pregnant after 12 months of unprotected intercourse. However, the use of MAR has moved beyond simply ‘curing’ a medical problem. Users of MAR now include gay and lesbian couples as well as single women and men who are not infertile according to the medical definition. Moreover, some people seek help before 12 months or even without any waiting period at all (Sanders *et al.*, 2014). People could know about their reduced chances of pregnancy and directly seek help without a period of trying, including those who have other medical problems or have received other treatments (e.g. cancer treatment), or those who have experienced problems before with another partner or when trying to become pregnant with their first child. Some biologically fertile women use ART as a means of conceiving in the absence of a male partner (Hertz, 2006). In addition, more lesbian women are choosing ART rather than ‘low-tech’ strategies as means to reproduce with their own eggs (Mamo, 2007). For gay couples, gestational surrogacy with egg donation and IVF is the only way of conceiving a child related to at least one partner. Including a measure of perceived fertility problems would provide additional value in some cases. Thus, it would also be a sensible approach to include all women or all men in the baseline. We do recommend that the decision is made explicit and the advantages and disadvantages of the chosen approach are discussed.

One other important factor that affects help-seeking rates is how the study population is defined. Sometimes the analytic sample is predefined by the population of the survey, but often researchers make choices during the process of preparing the data for analysis. For example, Stanford *et al.* (2016) reported a relatively high rate (59%) of medical help-seeking for US women, which can partly be explained by their choice of sample. It includes only married childless women aged 20–35 years who were trying to have a child for at least 12 months at the time of the interview; married women with no own children likely have strong intentions to have a child and thus will be more inclined to seek help. Yet another example is the study by Farland *et al.* (2016) whose even higher help-seeking rate is difficult to compare with other numbers for two reasons. First, medical help-seeking was inferred in a rather unusual way (Table I). Second, the study population is rather specific: because of their medical background, nurses might be more inclined to seek medical help than the general population. Consequently, researchers need to pay attention to characteristics of their study population such as marital status, parenthood, education/occupation and age.

### Medical help-seeking

Studies on the prevalence of MAR use do not usually provide formal definitions of medical help-seeking. Thus, we must infer what they mean by medical help-seeking by sometimes incomplete information presented in the studies. Some studies set the threshold for what is considered medical help-seeking very low, e.g. ‘consulted a doctor about problems conceiving’ (Oakley *et al.*, 2007). Other studies require ‘fertility evaluation or fertility treatment’ (Kessler *et al.*, 2013), and still others leave the decision as to what is considered as medical help-seeking up to the survey respondents by inquiring very generally if a person sought (medical) help to assist becoming pregnant (Olsen *et al.*, 1996; Marino *et al.*, 2011).

Selecting a cutoff point for discriminating between ‘help-seeking’ and ‘not help-seeking’ can have tremendous consequences for medical help-seeking rates. Not all receipt of medical advice concerning becoming pregnant is necessarily linked to MAR services; for example, a woman might merely ask a family physician what part of the menstrual cycle is the most fertile. On the other hand, advice on fertile windows might be the outcome of a medical help-seeking event to an IVF clinic. Thus, the broad questions some studies asked may produce misleading results. It could also be argued that merely receiving advice or talking to a doctor is not the same as receiving treatment.

It might therefore be preferable to conceptualize medical help-seeking as a continuum. Compared to studies that apply general or universal measures of medical help-seeking, studies that treat medical help-seeking as a process and differentiate different stages of medical help-seeking appear to be much more informative. It seems preferable to measure how far people move along the help-seeking continuum rather than choosing an arbitrary cutoff point. Studies that choose this more nuanced perspective are starting to accumulate as the majority of such articles have been published since 2010. The categories used in the NSFG and NSFB (described above) provide good examples. Studies treating medical help-seeking as a process have shown that, whereas a relatively large number at least talk to a doctor, the number of help-seekers decreases considerably for the use of more invasive treatments such as IVF. Thus, from the evidence presented for different countries, it becomes obvious that ART is the least frequently used treatment modality. This may possibly be because the majority of people can be helped with less invasive types of treatment. Some of those identified as infertile might become pregnant soon and therefore not need any help at all. But there might also be other social, cultural, or financial barriers to proceeding with diagnosis or getting certain types of treatments. As space does not permit a discussion of social, cultural and financial factors associated with medical help-seeking for infertility, we reserve that topic for a companion article.

### Additional remarks

We believe that the presentation of prevalence rates of MAR use should be improved upon. We found that only 4 of 30 papers on the prevalence of MAR use provide CIs; and only 3 of 15 papers on the stages of MAR use provide them. CIs inform us how precise prevalence rates are. Wide CIs indicate that a proportion is measured with low precision. We had aimed to calculate CIs for all prevalence rates to give readers the opportunity to judge their accuracy and how useful they are for practical applications. However, as several studies use complex survey data and apply weights, this was not feasible. A precondition for calculating CIs is that the sample is drawn randomly from a clearly specified population. This is not the case for all studies included here (e.g. column 3 in Tables I and II).

Collecting high-quality survey data is essential for reliable estimates of the prevalence of MAR use. Researchers must deal thoughtfully with issues such as sampling strategy, handling of unit and item non-response and the availability and use of weights. Note that Tables I and II do contain some basic information on the study design; however, a thorough discussion of these issues would have exceeded the scope of this article.

### Gaps in current research

By far the largest proportion of articles on medical help-seeking for infertility come from the USA, perhaps making our review appear ‘US-centric’. This is probably linked to the existence of the NSFG, a national demographic study that has measured infertility and MAR use for four decades. Thus, USA is the only country where it is possible to look at trends in medical help-seeking over a substantial period of time. Moreover, NSFG and the NSFB have the most elaborate indicators on the stages of help-seeking.

Even though the public awareness and the uptake of ART have increased over time (Calhaz-Jorge *et al.*, 2017), there does not seem to be strong evidence that the rate of medical help-seeking in general is increasing. Data from the USA suggest that the use of fertility treatment has become more stratified, with some women or couples receiving high-tech treatment while others receive only advice or nothing at all. If scholars are to develop a good sense of long-terms trends, we need to initiate similar studies in other countries as well. It is hard to generalize from the USA to other countries because of extreme differences in social and policy environments. USA is the only developed country without universal healthcare as well as a country where private insurance often provides only minimal coverage of MAR.

Most of the articles we reviewed have focused on a single country. However, careful country comparisons of prevalence rates can make different levels of help-seeking apparent and hence set the ground for further comparative research on the reasons for such differences. We are aware of only one attempt at comparing rates of MAR use from the early 1990s for five European countries, namely the European Studies of Infertility and Subfecundity (Olsen *et al.*, 1996; Küppers-Chinnow and Karmaus, 1997; Olsen *et al.*, 1998). Considerable effort was taken to achieve consistent definitions of infertility and medical help-seeking and comparable samples. We would like to see more such comparative studies for the above-mentioned reason. Europe, with its many countries and diversity of legislation and culture, is an ideal site to study the reasons for differences in the use of MAR. At the least, we suggest that single-country studies should routinely provide information on legal and cultural context, and findings should be interpreted against the backdrop of the country’s particular setting. When comparing findings from different studies, consideration of country context is crucial.

It is well known from demographic fertility research that both partners are involved in fertility decision-making. Even though evidence for men is starting to accumulate, quantitative studies with a gender-comparative perspective are still quite rare. For men, the medical help-seeking rates appear to be slightly lower compared to women (Anderson *et al.*, 2009; Hotaling *et al.*, 2012). At this time, it is unclear to what extent the gender differences found reflect real behavioral differences and how such differences might be interpreted. A recent study looked at the question of whether women are really better informants than their partners about received medical care for infertility: findings suggest that this is probably not true (Belgherbi and La Rochebrochard, 2018). Research on medical help-seeking for infertility will benefit from including men and couples in the analyses.

### Limitations

This review has several limitations. Although we believe strongly that a narrative review is preferable to a systematic review in this situation, narrative reviews provide great leeway for interpretation on the part of authors and therefore introduce the risk of bias. We believe that we have minimized the risk of bias by focusing primarily on conceptual and methodological differences between articles rather than on methodological evaluation. Still, it is possible that others might have looked at the same set of articles and drawn different conclusions.

One possible source of bias is publication bias, as we did limit our search to published articles. The threat of publication bias is perhaps curtailed here, however, because the studies reported here are population-based studies that require a major investment of resources, and such studies usually find publication outlets. Another possible source of bias involves language. We limited the literature search to languages the authors speak—English, French and German. It is thus possible—even likely—that we have overlooked articles written in other languages. However, we believe that, even if we have missed some sources, our analysis and critique remain valid.

It should be noted that some of the studies cited in this article were not carried out with the sole purpose of reporting the prevalence of medical help-seeking; rather, the chief purpose in some cases was to investigate the correlates of medical help-seeking. Thus, data may have been ‘cleaned’ to allow multivariable regression analysis, which means that list-wise deletion may have been applied before reporting the proportion of those seeking medical help. This multivariable regression procedure ensures that all analyses in a given study paper are based on the same sample size. If, however, missingness on the variables other than medical help-seeking itself is not random, medical help-seeking rates will be biased. None of the papers reviewed here used missing data imputation, which would likely generate more accurate estimates in such situations.

## Conclusion

In spite of the limitations, we believe that this review serves an important purpose by bringing together a diverse set of findings from different countries, by pointing to conceptual and methodological differences that make it difficult to compare studies, and by sketching out a way forward for the estimation of rates of MAR use. Careful consideration of methodological and conceptual issues involved in estimating the prevalence of MAR use is crucial if we are to make progress in this area.

## Authors’ roles

J.P-W. and A.L.G. designed the study. Both authors participated in the search for relevant papers for the review. J.P-W. was primarily responsible for writing the Introduction and Results sections and for constructing the tables. A.L.G. was primarily responsible for the Materials and Methods and Discussion. Both authors read, edited and approved all aspects of the final manuscript.

## Conflict of interest

None.
